# Outcomes of pregnancies complicated by maternal autoimmune diseases in Denmark, Finland, and Sweden: a multi-national population‐based register study

**DOI:** 10.1016/j.xagr.2026.100667

**Published:** 2026-06-20

**Authors:** Kelvin HM Kwok, Mika Gissler, Julie H Vendelbo, Rebecca Zaha, Sven Wegner, Yangjun Liu, Rafiq Muhammad, Vedran Stefanovic, Lars H Pedersen

**Affiliations:** a Ciencia Research AB, Stockholm, Sweden (Kwok, Liu, Muhammad); b Department of Data and Analytics, Finnish Institute for Health and Welfare, Helsinki, Finland (Gissler); c Department of Molecular Medicine and Surgery, Karolinska Institutet, Stockholm, Sweden (Gissler); d Department of Clinical Medicine, Aarhus University, Aarhus, Denmark (Vendelbo, Pedersen); e Johnson & Johnson, Spring House, PA, United States (Zaha); f Johnson & Johnson, Neuss, Germany (Wegner); g Department of Obstetrics and Gynecology, Fetomaternal Medical Center, Helsinki University Hospital and University of Helsinki, Helsinki, Finland (Stefanovic); h Department of Obstetrics and Gynecology, Aarhus University Hospital, Aarhus, Denmark (Pedersen)

**Keywords:** autoimmune diseases, pregnancy outcomes, neonatal health, population-based study, Sjögren’s disease, systemic lupus erythematosus, rheumatoid arthritis, idiopathic inflammatory myopathies, mixed connective tissue disease

## Abstract

**Background:**

Maternal autoimmune diseases, including Sjögren’s disease (SjD), systemic lupus erythematosus (SLE), rheumatoid arthritis (RA), idiopathic inflammatory myopathies (IIM), and mixed connective tissue disease (MCTD), are associated with adverse pregnancy outcomes. However, comprehensive population-based studies evaluating the impact of these diseases across multiple countries are limited.

**Objective:**

To characterize outcomes in pregnancies complicated by SjD, SLE, RA, IIM, and/or MCTD in Denmark, Finland, and Sweden.

**Study Design:**

A population-based cohort study was conducted using data from national health registers from Denmark (1995–2017), Finland (2000–2022), and Sweden (2000–2021). The study included singleton pregnancies in women aged ≥15 years at delivery and with any of the five specified autoimmune diseases diagnosed before or during pregnancy, or within one year postpartum. Maternal demographics and complications, birth outcomes, neonatal characteristics, and long-term outcomes in children were described.

**Results:**

The present study included 20,425 singleton pregnancies complicated by maternal SjD (n=1913), SLE (n=3570), RA (n=13,328), IIM (n=1078), or MCTD (n=536). Across the three countries, common pregnancy complications included preeclampsia and preterm premature rupture of membranes, which occurred in 2% to 12%, and 1% to 16% of pregnancies, respectively; fetal growth restriction was reported in 3% to 11% of pregnancies complicated by SjD, SLE, RA, or MCTD. Emergency caesarean section was performed in 10% to 21% of all pregnancies. A total of 20,304 liveborn children were identified, of which 6% to 18% were preterm and 5% to 24% were small for gestational age. Neonatal unit admission rate varied between 9% and 24%. Second/third-degree atrioventricular block was reported in 1.5% and 0.9% of SjD pregnancies in Sweden and Finland, respectively.

**Conclusion:**

The study provides a comprehensive overview of outcomes in pregnancies complicated by autoimmune diseases in three Nordic countries. These pregnancies show notable proportions of adverse maternal and neonatal outcomes, underscoring the importance of tailored clinical management and specialized perinatal care to address the unique challenges faced by the mothers and their children.

**AJOG Global Reports at a Glance**

**A. Why was the study conducted?**

To characterize real-world outcomes in pregnancies complicated by maternal autoimmune diseases in Denmark, Finland, and Sweden.

**B. What are the key findings?**

Pregnancies complicated by SjD, SLE, RA, IIM, and/or MCTD showed notable risks of adverse maternal and neonatal outcomes, including preeclampsia, fetal growth restriction, emergency caesarean section, and neonatal unit admission.

**C. What does this study add to what is known?**

This study provides a comprehensive overview of outcomes in pregnancies complicated by autoimmune diseases across three Nordic countries, underscoring an ongoing unmet medical need. These insights lay the foundation for improving healthcare support and developing targeted interventions to address the unique challenges faced by pregnant women with autoimmune disorders.

## Introduction

Autoimmune diseases comprise a diverse group of disorders^1^, ^2^, ^3^, ^4^ such as Sjögren’s disease (SjD), systemic lupus erythematosus (SLE), rheumatoid arthritis (RA), idiopathic inflammatory myopathies (IIM), and mixed connective tissue disease (MCTD) with significant impact on quality of life and life expectancy. These disorders disproportionately affect women of reproductive age, posing potential risks to maternal and fetal health.

Pregnancies complicated by autoimmune diseases are associated with increased risk of complications such as preeclampsia, preterm birth, stillbirth, emergency caesarean section, low birth weight, small for gestational age (SGA), congenital anomalies, and neonatal lupus.^5^, ^6^, ^7^, ^8^, ^9^, ^10^, ^11^, ^12^, ^13^ Specific maternal autoantibodies can be problematic in pregnancy; e.g., anti-phospholipid (aPL) antibodies increases the risk of placental complications including preeclampsia,^14^ and anti-Ro/SSA and anti-La/SSB specifically cause congenital heart block.^15^ As a result, international guidelines recommend testing for autoantibodies in women with SjD or SLE to better assess pregnancy risks.^16^, ^17^, ^18^ However, these guidelines often lack details, such as the criteria and schedule for testing of at-risk patients and the recommended test assays,^19^, ^20^, ^21^ which hinders standardized implementation. This highlights the complex management of pregnancies complicated by autoimmune diseases, and the need to improve our understanding of associated risks. Research on pregnancy outcomes in women with autoimmune diseases is often constrained by small sample sizes, single-country cohorts, or a lack of population-wide data.^22^, ^23^, ^24^, ^25^, ^26^, ^27^ Such evidence gap makes it challenging to develop unified and detailed guidelines.^28^, ^29^, ^30^ A more comprehensive understanding of these risks in larger, more generalizable cohorts is critical for informing clinical care and improving outcomes.

To address this, the present study leverages nationwide population-based health registers from Denmark, Finland, and Sweden to characterize pregnancies in women with SjD, SLE, RA, IIM, and MCTD. By incorporating data from three Nordic countries and applying consistent analytic methods across all settings, this study aims to provide a uniquely robust and comprehensive overview of adverse pregnancy outcomes associated with maternal autoimmune conditions, with the goal of informing evidence-based guidelines and improving clinical management.

## Materials and methods

### Study design and data sources

This was a population-based cohort study using data from national health registers in Denmark, Finland, and Sweden. In Denmark, data were obtained from the National Patient Register, Medical Birth Register, Cause of Death Register, and National Prescription Database. In Finland, data were obtained from the Care Register for Health Care, Medical Birth Register, Cause of Death Register, and Register on Reimbursed Medication. In Sweden, data were obtained from the National Patient Register, Medical Birth Register, Cause of Death Register, Prescribed Drug Register, and Swedish Neonatal Quality Register. Within each country, individual-level data were linked between national registers based on the mother’s and newborn’s unique identification numbers. Detailed descriptions of the specific registers used are provided in the Supplementary Method.

### Study population

This study included pregnancies in women who were diagnosed with SjD, SLE, RA, IIM, or MCTD prior to delivery date, or no later than one year postpartum to capture any preclinical disease that may be present during pregnancy. A given woman could contribute more than one pregnancy if multiple pregnancies met the inclusion criteria. The 10th version of International Statistical Classification of Diseases and Related Health Problems (ICD-10) codes used to identify the autoimmune diseases of interest are listed in the Supplementary Table 1. A confirmed diagnosis of autoimmune disease was defined as at least two outpatient or one inpatient visit with the respective ICD-10 code registered as the primary or secondary diagnosis by specialists. If multiple autoimmune diseases were identified in the same pregnant woman, the earliest diagnosis was considered.

The study cohort included women who were diagnosed with SjD, SLE, RA, IIM, or MCTD, their singleton pregnancies, and neonates from these pregnancies, provided that the autoimmune disease diagnoses and the deliveries fell within the pre-defined study periods (Denmark: 1995–2017 for diagnosis, 1997–2017 for deliveries; Finland: 2000–2022 for both; Sweden: 2000–2021 for both). Pregnancies were excluded if the mother was younger than 15 years at delivery or if the earliest recorded autoimmune disease diagnosis occurred more than one year after delivery. Pregnancies that ended before 22 weeks of gestation (or 28 weeks of gestation prior to 1 April 2004 in Denmark and 1 July 2008 in Sweden) were not registered at the national Medical Birth Registers of the countries examined and were therefore excluded from this study.

### Outcomes

Maternal characteristics and complications during pregnancy were described. Outcomes of livebirths occurred during neonatal period and conditions diagnosed beyond neonatal period, as well as mortality and autoimmune atrioventricular block diagnosed during the entire follow-up were examined. Most maternal, neonatal, and childhood conditions were based on diagnosis in the health registers, all related ICD-10 codes are presented in Supplementary Table 2. Fetal growth restriction (FGR) was defined by the ICD-10 code O36.5, whereas SGA was defined as birth weight <10th percentile of expected gestational age based on the estimated fetal weight chart established by Maršál et al.^31^ Preterm birth was defined as live births delivered before 37 weeks of gestational age. Low birth weight was defined as birth weight below 2500 g.

### Statistical analysis

Descriptive statistics were used to summarize maternal and neonatal characteristics and pregnancy outcomes. Continuous variables were summarized using mean, median, standard deviation (SD), and interquartile range (IQR). Categorical variables were summarized using frequency and percentage. Data from each country were analyzed separately, and results were presented by autoimmune disease type and country. Values less than 5 are reported as “<5” to ensure data anonymity according to national data protection rules and regulations. All analyses were conducted using statistical software R and SAS 9.4.

### Ethical approval

This study was approved by the Danish Data Protection Agency (AU 2016-051-000001, lbnr. 2227), Finnish Social and Health Data Permit Authority (Findata, THL/4252/14.02.00/2022), and Swedish Ethical Review Authority (Dnr2022-02995-01). Individual informed consent was not required for register-based study when the data are pseudonymized and the registered persons are not contacted.

## Results

### Study cohort

The study included 12,680 women and 20,425 singleton pregnancies across the three Nordic countries: 3312 women with 5968 pregnancies in Denmark, 3394 women with 5323 pregnancies in Finland, and 5974 women with 9134 pregnancies in Sweden. Table 1 displays detailed characteristics of the study cohort. The distribution of maternal autoimmune diseases was similar across countries, with RA being the most prevalent (64%–67% of all pregnancies included) and MCTD the least common (2%–4%). In Denmark and Sweden, SLE was more prevalent than SjD, while proportions of SLE and SjD were comparable in Finland. In addition, the mean age at earliest autoimmune disease diagnosis was older in Finland (32 years) compared to Sweden (29 years) and Denmark (28 years).

**Table 1:** Characteristics of study cohort

	Overall	Denmark	Finland	Sweden
Pregnant women, n (%)				
Total	12680	3312	3394	5974
SjD	1224 (9.7)	203 (6.1)	486 (14.3)	535 (9.0)
SLE	2219 (17.5)	527 (15.9)	456 (13.4)	1236 (20.7)
RA	8283 (65.3)	2222 (67.1)	2239 (66.0)	3822 (64.0)
IIM	628 (5.0)	293 (8.9)	97 (2.9)	238 (4.0)
MCTD	326 (2.6)	67 (2.0)	116 (3.4)	143 (2.4)
Age (years) at earliest diagnosis of autoimmune disease				
Mean (SD)	29.2 (6.3)	27.6 (6.5)	31.9 (5.1)	28.6 (6.3)
Singleton pregnancies, n (%)				
Total	20425	5968	5323	9134
SjD	1913 (9.4)	380 (6.4)	748 (14.1)	785 (8.6)
SLE	3570 (17.5)	923 (15.5)	723 (13.6)	1924 (21.1)
RA	13328 (65.3)	4014 (67.3)	3499 (65.7)	5815 (63.7)
IIM	1078 (5.3)	529 (8.9)	165 (3.1)	384 (4.2)
MCTD	536 (2.6)	122 (2.0)	188 (3.5)	226 (2.5)
Note: For women and pregnancies with multiple autoimmune diseases, only the earliest autoimmune disease was counted. *SjD*, Sjögren’s disease; *SLE*, systemic lupus erythematosus; *RA*, rheumatoid arthritis; *IIM*, idiopathic inflammatory myopathy; *MCTD*, mixed connective tissue disease; *SD*, standard deviation. *Kwok et al. Outcomes of pregnancies complicated by maternal autoimmune diseases in Denmark, Finland, and Sweden: a multi-national population‐based register study. Am J Obstet Gynecol 2026*.				

### Maternal characteristics

Maternal characteristics of pregnant women with autoimmune disease are presented in Table 2. Overall, the mean maternal age at delivery ranged between 30 and 33 years. Parity distribution was comparable across the autoimmune diseases and across countries, with 36% to 50% of pregnancies being the first parity and 26% to 39% of pregnancies being the second parity (Table 2). At the time of registration to antenatal care, most pregnant women had a normal BMI (41%–66%).

**Table 2:** Maternal characteristics

	SjD (n=1913)			SLE (n=3570)			RA (n=13,328)			IIM (n=1078)			MCTD (n=536)		
	Denmark	Finland	Sweden	Denmark	Finland	Sweden	Denmark	Finland	Sweden	Denmark	Finland	Sweden	Denmark	Finland	Sweden
Singleton pregnancies	380	748	785	923	723	1924	4014	3499	5815	529	165	384	122	188	226
Age at delivery (years)															
Mean (SD)	31.5 (5.1)	33.1 (4.7)	33.2 (5.0)	31.2 (4.8)	32 (5.0)	32.0 (4.7)	31.0 (5.1)	32.4 (5.0)	32.7 (4.8)	30.4 (5.1)	31 (5.5)	30.6 (5.1)	30.8 (4.6)	31.3 (4.8)	31.2 (4.6)
Median (IQR)	31 (28-35)	33 (30-36)	33 (30-36)	31 (28-35)	32 (29-35)	32 (29-35)	31 (28-35)	33 (29-36)	33 (29-36)	30 (27-34)	31 (28-34)	31 (27-34)	31 (28-34)	32 (29-35)	31 (28-34)
Parity, n (%)															
1	168 (44.2)	268 (35.8)	314 (40.0)	454 (49.2)	298 (41.2)	837 (43.5)	1912 (47.6)	1347 (38.5)	2442 (42.0)	233 (44.0)	64 (38.8)	180 (46.9)	59 (48.4)	86 (45.7)	112 (49.6)
2	136 (35.8)	268 (35.8)	295 (37.6)	312 (33.8)	260 (36.0)	713 (37.1)	1415 (35.3)	1226 (35.0)	2249 (38.7)	186 (35.2)	42 (25.5)	136 (35.4)	45 (36.9)	67 (35.6)	83 (36.7)
3	54 (14.2)	136 (18.2)	115 (14.7)	102 (11.1)	101 (14.0)	256 (13.3)	471 (11.7)	537 (15.3)	769 (13.2)	63 (11.9)	24 (14.5)	54 (14.1)	12 (9.8)	26 (13.8)	25 (11.1)
4	10 (2.6)	59 (7.9)	41 (5.2)	34 (3.7)	44 (6.1)	78 (4.1)	125 (3.1)	262 (7.5)	227 (3.9)	24 (4.5)	14 (8.5)	≤10 (≤2.6)	<5 (<4.1)	<10 (<5.3)	≤5 (≤2.2)
≥5	8 (2.1)	17 (2.3)	20 (2.6)	13 (1.4)	20 (2.8)	40 (2.1)	56 (1.4)	125 (3.6)	128 (2.2)	19 (3.6)	21 (12.7)	≤10 (≤2.6)	<5 (<4.1)	<5 (<2.7)	≤5 (≤2.2)
Missing	<5 (<1.3)	0 (0)	0 (0)	8 (0.9)	0 (0)	0 (0)	35 (0.9)	0 (0)	0 (0)	<5 (<0.9)	0 (0)	0 (0)	<5 (<4.1)	0 (0)	0 (0)
Body mass index upon registration at antenatal care, n (%)															
Underweight (<18.5)	22 (5.8)	23 (3.1)	21 (2.7)	35 (3.8)	20 (2.8)	59 (3.1)	121 (3)	109 (3.1)	139 (2.4)	26 (4.9)	<5 (<3)	11 (2.9)	<5 (<4.1)	<5 (<2.7)	7 (3.1)
Normal weight (18.5-24.9)	186 (48.9)	370 (49.5)	472 (60.1)	396 (42.9)	359 (49.7)	1041 (54.1)	1725 (43)	1732 (49.5)	3127 (53.8)	215 (40.6)	87 (52.7)	208 (54.2)	66 (54.1)	108 (57.4)	148 (65.5)
Overweight (25.0-29.9)	25 (6.6)	156 (20.9)	165 (21.0)	82 (8.9)	130 (18)	433 (22.5)	411 (10.2)	732 (20.9)	1379 (23.7)	46 (8.7)	34 (20.6)	92 (24.0)	14 (11.5)	33 (17.6)	34 (15.0)
Obese (≥30.0)	49 (12.9)	78 (10.4)	68 (8.7)	153 (16.6)	63 (8.7)	212 (11.0)	655 (16.3)	489 (14)	741 (12.7)	83 (15.7)	21 (12.7)	47 (12.2)	16 (13.1)	20 (10.6)	16 (7.1)
Missing	98 (25.8)	121 (16.2)	59 (7.5)	257 (27.8)	151 (20.9)	179 (9.3)	1102 (27.5)	437 (12.5)	429 (7.4)	159 (30.1)	<20 (<12.1)	26 (6.8)	<30 (<24.6)	<20 (<10.6)	21 (9.3)

Note: *SjD*, Sjögren’s disease; *SLE*, systemic lupus erythematosus; *RA*, rheumatoid arthritis; *IIM*, idiopathic inflammatory myopathy; *MCTD*, mixed connective tissue disease; *SD*, standard deviation; *IQR*: interquartile range. To preserve patient anonymity, values less than 5 were presented as ‘<5’, and other values that may reveal the small number were presented as a range.

*Kwok et al. Outcomes of pregnancies complicated by maternal autoimmune diseases in Denmark, Finland, and Sweden: a multi-national population‐based register study. Am J Obstet Gynecol 2026.*

### Pregnancy outcomes

Table 3 summarizes pregnancy complications and outcomes across autoimmune disease groups and countries. Several complications were commonly reported across disease groups, including preeclampsia (2%–12%), gestational hypertension (1%–7%), FGR (≤11%), gestational diabetes (≤17%, with higher prevalence generally observed in Finland), emergency caesarean section (10%–21%), and PPROM (1%–16%). Stillbirth occurred in fewer than 3% of pregnancies across all groups, and in some disease groups and countries, none or fewer than five cases were recorded. Other complications such as eclampsia, placental abruption, placenta previa, proteinuria, and obstetric embolism occurred infrequently across all groups.

**Table 3:** Pregnancy outcomes

	SjD (n=1913)			SLE (n=3570)			RA (n=13,328)			IIM (n=1078)			MCTD (n=536)		
	Denmark	Finland	Sweden	Denmark	Finland	Sweden	Denmark	Finland	Sweden	Denmark	Finland	Sweden	Denmark	Finland	Sweden
Singleton pregnancies	380	748	785	923	723	1924	4014	3499	5815	529	165	384	122	188	226
Pregnancy complications, n (%)															
Preeclampsia	16 (4.2)	17 (2.3)	25 (3.2)	92 (10.0)	47 (6.5)	156 (8.1)	191 (4.8)	128 (3.7)	255 (4.4)	19 (3.6)	5 (3.0)	19 (5.0)	11 (9.0)	10 (5.3)	26 (11.5)
Eclampsia	0 (0)	0 (0)	0 (0)	<5 (<0.5)	0 (0)	<5 (<0.3)	<5 (<0.1)	<5 (<0.1)	<5 (<0.1)	0 (0)	0 (0)	0 (0)	0 (0)	0 (0)	0 (0)
Gestational hypertension	<5 (<1.3)	49 (6.6)	15 (1.9)	30 (3.3)	36 (5.0)	33 (1.7)	113 (2.8)	106 (3.0)	109 (1.9)	12 (2.3)	12 (7.3)	5 (1.3)	<5 (<4.1)	10 (5.3)	6 (2.7)
Gestational diabetes	5 (1.3)	103 (13.8)	9 (1.2)	30 (3.3)	90 (12.4)	33 (1.7)	110 (2.7)	604 (17.3)	107 (1.8)	24 (4.5)	23 (13.9)	<5 (<1.3)	0 (0)	21 (11.2)	<5 (<2.2)
PPROM	33 (8.7)	25 (3.3)	11 (1.4)	53 (5.7)	26 (3.6)	75 (3.9)	322 (8.0)	173 (4.9)	176 (3.0)	35 (6.6)	9 (5.5)	9 (2.3)	19 (16.0)	11 (5.9)	9 (4.0)
Placental abruption	<5 (<1.3)	<5 (<0.7)	<5 (<0.6)	10 (1.1)	<5 (<0.7)	11 (0.6)	35 (0.9)	<5 (<0.1)	25 (0.4)	7 (1.3)	<5 (<3)	<5 (<1.3)	<5 (<4.1)	<5 (<2.7)	<5 (<2.2)
Placenta previa	<5 (<1.3)	8 (1.1)	3 (0.4)	10 (1.1)	6 (0.8)	14 (0.7)	47 (1.2)	41 (1.2)	31 (0.5)	<5 (<0.9)	<5 (<3)	<5 (<1.3)	<5 (<4.1)	<5 (<2.7)	<5 (<2.2)
Proteinuria during pregnancy	<5 (<1.3)	<5 (<0.7)	<5 (<0.6)	14 (1.5)	6 (0.8)	<5 (<0.3)	18 (0.4)	8 (0.2)	6 (0.1)	<5 (<0.9)	0 (0)	0 (0)	0 (0)	<5 (<2.7)	<5 (<2.2)
Obstetric embolism	<5 (<1.3)	0 (0)	0 (0)	<5 (<0.5)	0 (0)	<5 (<0.3)	<5 (<0.1)	0 (0)	<5 (<0.1)	<5 (<0.9)	0 (0)	0 (0)	<5 (<4.1)	0 (0)	0 (0)
FGR	21 (5.5)	41 (5.5)	25 (3.2)	99 (11.0)	49 (6.8)	86 (4.5)	185 (4.6)	128 (3.7)	155 (2.7)	19 (3.6)	<5 (<3)	<5 (<1.3)	6 (4.9)	14 (7.4)	9 (4.0)
Mode of delivery, n (%)															
Vaginal	290 (76.3)	553 (73.9)	573 (73.0)	587 (63.6)	522 (72.2)	1350 (70.2)	2957 (73.7)	2733 (78.1)	4339 (74.6)	405 (76.6)	129 (78.2)	294 (76.6)	85 (69.7)	140 (74.5)	157 (69.5)
Caesarean	90 (23.7)	195 (26.1)	212 (27.0)	335 (36.3)	201 (27.8)	574 (29.8)	1046 (26.1)	762 (21.8)	1476 (25.4)	121 (22.9)	36 (21.8)	90 (23.4)	36 (29.5)	48 (25.5)	69 (30.5)
Elective	32 (8.4)	99 (13.2)	105 (13.4)	141 (15.3)	73 (10.1)	219 (11.4)	401 (10.0)	315 (9.0)	715 (12.3)	52 (9.8)	12 (7.3)	42 (10.9)	12 (9.8)	9 (4.8)	28 (12.4)
Emergency	45 (11.8)	96 (12.8)	98 (12.5)	139 (15.1)	128 (17.7)	323 (16.8)	500 (12.5)	447 (12.8)	680 (11.7)	55 (10.4)	24 (14.5)	43 (11.2)	20 (16.4)	39 (20.7)	32 (14.2)
Missing caesarean type	13 (3.4)	0 (0)	8 (1.0)	55 (6.0)	0 (0)	32 (1.7)	145 (3.6)	0 (0)	76 (1.3)	14 (2.6)	0 (0)	5 (1.3)	<5 (<4.1)	0 (0)	9 (4.0)
Missing	0 (0)	0 (0)	<5 (<0.6)	<5 (<0.5)	0 (0)	0 (0)	11 (0.3)	<5 (<0.1)	5 (0.1)	<5 (<0.9)	0 (0)	0 (0)	<5 (<4.1)	0 (0)	0 (0)
Birth outcome, n (%)															
Stillbirth	<5 (<1.3)	8 (1.1)	5 (0.6)	21 (2.3)	<5 (<0.7)	15 (0.8)	16 (0.4)	11 (0.3)	27 (0.5)	<5 (<0.9)	<5 (<3)	<5 (<1.3)	0 (0)	<5 (<2.7)	0 (0)

Note: *SjD*, Sjögren’s disease; *SLE*, systemic lupus erythematosus; *RA*, rheumatoid arthritis; *IIM*, idiopathic inflammatory myopathy; *MCTD*, mixed connective tissue disease; *SD*, standard deviation; *IQR*: interquartile range; *PPROM*, premature rupture of membranes; *FGR*, fetal growth restriction. To preserve patient anonymity, values less than 5 were presented as '<5′, and others that may reveal the small number were presented as a range.

*Kwok et al. Outcomes of pregnancies complicated by maternal autoimmune diseases in Denmark, Finland, and Sweden: a multi-national population‐based register study. Am J Obstet Gynecol 2026.*

### Neonatal and long-term outcomes

A total of 20,304 singleton newborns were identified in this study, including 5295 from Finland, 5924 from Denmark, and 9085 from Sweden. The overall sex ratio was balanced across disease groups and countries (Table 4). Neonatal and long-term outcomes of these newborns are summarized in Table 4, Table 5.

**Table 4:** Neonatal characteristics of liveborn children

	SjD (n=1896)			SLE (n=3530)			RA (n=13,274)			IIM (n=1072)			MCTD (n=532)		
	Denmark	Finland	Sweden	Denmark	Finland	Sweden	Denmark	Finland	Sweden	Denmark	Finland	Sweden	Denmark	Finland	Sweden
Liveborn children, n (%)	376	740	780	902	719	1909	3998	3488	5788	526	164	382	122	184	226
Sex, n (%)															
Female	194 (52)	365 (49.3)	371 (47.6)	439 (49)	362 (50.3)	935 (49.0)	1976 (49)	1695 (48.6)	2856 (49.3)	244 (46)	74 (45.1)	192 (50.3)	59 (48)	89 (48.4)	106 (46.9)
Gestational age (weeks), n (%)															
Mean (SD)	39.4 (1.9)	39.3 (2.0)	39 (2.0)	38.2 (3.4)	38.7 (2.6)	38.2 (2.7)	39.5 (2.0)	39.5 (1.9)	38.9 (2.1)	39.5 (2.0)	39.4 (2.0)	38.8 (2.2)	38.7 (3.0)	38.7 (2.7)	38.3 (2.4)
Median (IQR)	40 (39-41)	40 (39-41)	39 (38-40)	39 (38-40)	39 (38-40)	39 (37-40)	40 (39-41)	40 (39-41)	39 (38-40)	40 (39-41)	40 (38-41)	39 (38-40)	39 (38-40)	39 (38-40)	39 (37-40)
<37 weeks	29 (7.7)	61 (8.2)	60 (7.7)	159 (17.6)	108 (15.0)	276 (14.5)	285 (7.1)	259 (7.4)	479 (8.3)	32 (6.1)	13 (7.9)	36 (9.4)	18 (14.8)	26 (14.1)	32 (14.2)
37-38 weeks	103 (27.0)	176 (23.8)	193 (24.7)	303 (34.0)	198 (27.5)	587 (30.7)	891 (22.0)	745 (21.4)	1523 (26.3)	130 (25.0)	40 (24.4)	101 (26.4)	28 (23.0)	50 (27.2)	71 (31.4)
39-40 weeks	171 (45.0)	384 (51.9)	373 (47.8)	325 (36.0)	331 (46.0)	810 (42.4)	1911 (48.0)	1801 (51.6)	2607 (45.0)	257 (49.0)	80 (48.8)	178 (46.6)	59 (48.0)	83 (45.1)	96 (42.5)
≥41 weeks	73 (19.0)	118 (15.9)	153 (19.6)	115 (13.0)	82 (11.4)	234 (12.3)	911 (23.0)	677 (19.4)	1177 (20.3)	107 (20.0)	31 (18.9)	67 (17.5)	17 (14.0)	25 (13.6)	27 (11.9)
Missing	0 (0)	<5 (<0.7)	<5 (<0.6)	0 (0)	0 (0)	<5 (<0.3)	0 (0)	<10 (<0.3)	<5 (<0.1)	0 (0)	0 (0)	0 (0)	0 (0)	0 (0)	0 (0)
Birth weight (g), n (%)															
Mean (SD)	3329 (536)	3353 (589)	3323 (577)	3090 (800)	3179 (691)	3196 (696)	3447 (597)	3457 (564)	3429 (598)	3455 (564)	3422 (579)	3445 (606)	3061 (678)	3128 (703)	3160 (638)
Median (IQR)	3337 (3000-3698)	3390 (3082-3730)	3355 (3047-3670)	3210 (2767-3580)	3238 (2846-3600)	3270 (2866-3630)	3480 (3110-3830)	3485 (3150-3820)	3456 (3098-3809)	3457 (3106-3820)	3403 (3093-3870)	3465 (3118-3820)	3185 (2768-3463)	3208 (2825-3606)	3195 (2790-3530)
<2500	19 (5.1)	50 (6.8)	57 (7.3)	150 (17.0)	98 (13.6)	221 (11.6)	220 (5.5)	161 (4.6)	323 (5.6)	18 (3.4)	9 (5.5)	23 (6.0)	16 (13.0)	26 (14.1)	28 (12.4)
2500-3999	319 (85.0)	608 (82.2)	649 (83.2)	668 (74.0)	550 (76.5)	1511 (79.2)	3105 (78.0)	2809 (80.5)	4581 (79.1)	425 (8.1)	129 (78.7)	300 (78.5)	98 (80.0)	146 (79.3)	185 (81.9)
≥4000	36 (10.0)	81 (10.9)	73 (9.4)	77 (8.5)	71 (9.9)	174 (9.1)	650 (16.0)	516 (14.8)	876 (15.1)	81 (15.0)	26 (15.9)	58 (15.2)	6 (4.9)	12 (6.5)	12 (5.3)
Missing	<5 (<1.3)	<5 (<0.7)	<5 (<0.6)	7 (0.8)	0 (0)	<5 (<0.3)	23 (0.6)	<5 (<0.1)	8 (0.1)	<5 (<1)	0 (0)	<5 (<1.3)	<5 (<4.1)	0 (0)	<5 (<2.2)
Birth length (cm)															
Mean (SD)	51.1 (2.7)	49.6 (2.9)	49.7 (2.7)	50.0 (4.8)	48.8 (3.6)	49.1 (3.5)	51.6 (3.0)	49.9 (2.4)	50 (2.7)	51.6 (2.5)	49.7 (2.8)	49.9 (2.7)	49.9 (4.1)	48.7 (3.5)	49.1 (3.1)
Apgar score, n (%)															
1 min															
Mean (SD)	NA	8.4 (1.4)	8.8 (1.0)	NA	8.4 (1.5)	8.6 (1.5)	NA	8.6 (1.2)	8.7 (1.2)	NA	8.6 (1.2)	8.7 (1.3)	NA	8.2 (1.6)	8.6 (1.5)
<7	NA	57 (7.7)	35 (4.5)	NA	61 (8.5)	163 (8.5)	NA	187 (5.4)	317 (5.5)	NA	7 (4.3)	24 (6.3)	NA	19 (10.3)	14 (6.2)
7-10	NA	681 (92.0)	741 (95.0)	NA	656 (91.2)	1737 (91.0)	NA	3288 (94.3)	5445 (94.1)	NA	157 (95.7)	356 (93.2)	NA	165 (89.7)	212 (93.8)
Missing	NA	<5 (<0.7)	<5 (<0.6)	NA	<5 (<0.7)	9 (0.5)	NA	13 (0.4)	26 (0.4)	NA	0 (0)	<5 (<1.3)	NA	0 (0)	0 (0)
5 min															
Mean (SD)	9.8 (0.7)	8.9 (1.1)	9.7 (0.7)	9.7 (1.1)	8.8 (1.2)	9.6 (1.0)	9.9 (0.7)	9.0 (0.9)	9.7 (0.8)	9.8 (0.7)	9.1 (0.8)	9.7 (0.9)	9.8 (0.9)	8.9 (1.0)	9.7 (0.9)
<7	<5 (<1.3)	7 (0.9)	10 (1.3)	18 (2.0)	8 (1.1)	48 (2.5)	30 (0.8)	37 (1.1)	88 (1.5)	5 (1.0)	<5 (<3)	7 (1.8)	<5 (<4.1)	<5 (<2.7)	5 (2.2)
7-10	370 (98.4)	679 (91.8)	766 (98.2)	874 (96.9)	656 (91.2)	1853 (97.1)	3944 (98.6)	3286 (94.2)	5672 (98.0)	519 (98.7)	157 (95.7)	373 (97.6)	118 (96.7)	165 (89.7)	221 (97.8)
Missing	<5 (<1.3)	54 (7.3)	<5 (<0.6)	10 (1.1)	55 (7.6)	8 (0.4)	24 (0.6)	165 (4.7)	28 (0.5)	<5 (<1)	6 (3.7)	<5 (<1.3)	<5 (<4.1)	16 (8.7)	0 (0)
Note: *SjD*, Sjögren’s disease; *SLE*, systemic lupus erythematosus; *RA*, rheumatoid arthritis; *IIM*, idiopathic inflammatory myopathy; *MCTD*, mixed connective tissue disease; *SD*, standard deviation; *IQR*: interquartile range. *NA*: not available. To preserve patient anonymity, values less than 5 were presented as '<5′, and others that may reveal the small number were presented as a range. *Kwok et al. Outcomes of pregnancies complicated by maternal autoimmune diseases in Denmark, Finland, and Sweden: a multi-national population‐based register study. Am J Obstet Gynecol 2026.*

**Table 5:** Neonatal and long-term outcomes of liveborn children

	SjD (n=1896)			SLE (n=3530)			RA (n=13,274)			IIM (n=1072)			MCTD (n=532)		
	Denmark	Finland	Sweden	Denmark	Finland	Sweden	Denmark	Finland	Sweden	Denmark	Finland	Sweden	Denmark	Finland	Sweden
Liveborn children, n (%)	376	740	780	902	719	1909	3998	3488	5788	526	164	382	122	184	226
Conditions ≤28 days after birth, n (%)															
Admission to neonatal unit	47 (13.0)	144 (19.5)	71 (9.1)	175 (19.0)	156 (21.7)	252 (13.2)	435 (11.0)	514 (14.7)	520 (9.0)	53 (10.0)	23 (14.0)	44 (11.5)	19 (16.0)	44 (23.9)	29 (12.8)
SGA	67 (17.8)	93 (12.6)	100 (12.8)	194 (21.5)	124 (17.2)	281 (14.7)	521 (13.0)	335 (9.6)	544 (9.4)	68 (12.9)	10 (6.1)	19 (5.0)	29 (23.8)	41 (22.3)	39 (17.3)
FGR-associated SGA	7 (1.9)	23 (3.1)	20 (2.6)	58 (6.4)	30 (4.2)	71 (3.7)	84 (2.1)	63 (1.8)	125 (2.2)	8 (1.5)	<5 (<3)	<5 (<1.3)	<5 (<4.1)	10 (5.4)	9 (4.0)
Congenital anomalies	26 (6.9)	50 (6.8)	47 (6.0)	70 (7.8)	44 (6.1)	71 (3.7)	210 (5.3)	191 (5.5)	249 (4.3)	28 (5.3)	10 (6.1)	11 (2.9)	10 (8.2)	12 (6.5)	14 (6.2)
Cutaneous lupus	0 (0)	0 (0)	<5 (<0.6)	5 (0.6)	0 (0)	0 (0)	<5 (<0.1)	<5 (<0.1)	<5 (<0.1)	0 (0)	0 (0)	0 (0)	0 (0)	0 (0)	0 (0)
Neonatal jaundice	28 (7.4)	26 (3.5)	22 (2.8)	103 (11.0)	58 (8.1)	139 (7.3)	266 (6.7)	169 (4.8)	290 (5.0)	32 (6.1)	11 (6.7)	16 (4.2)	6 (4.9)	9 (4.9)	14 (6.2)
Respiratory distress	32 (8.5)	34 (4.6)	36 (4.6)	91 (10.0)	39 (5.4)	126 (6.6)	264 (6.6)	147 (4.2)	240 (4.1)	33 (6.3)	5 (3.0)	16 (4.2)	11 (9.0)	13 (7.1)	11 (4.9)
Hypoglycemia	18 (4.8)	21 (2.8)	19 (2.4)	61 (6.8)	25 (3.5)	79 (4.1)	188 (4.7)	117 (3.4)	164 (2.8)	28 (5.3)	<10 (<6.1)	11 (2.9)	<5 (<4.1)	<5 (<2.7)	10 (4.4)
Birth asphyxia	19 (5.1)	15 (2.0)	7 (0.9)	60 (6.7)	16 (2.2)	27 (1.4)	193 (4.8)	48 (1.4)	47 (0.8)	17 (3.2)	<5 (<3)	<5 (<1.3)	9 (7.4)	<10	<5 (<2.2)
Infections	<5 (<1.3)	<5 (<0.7)	9 (1.2)	5 (0.6)	0 (0)	26 (1.4)	9 (0.2)	0 (0)	74 (1.3)	5 (1.0)	0 (0)	8 (2.1)	<5 (<4.1)	0 (0)	<5 (<2.2)
Meconium aspiration	<5 (<1.3)	<5 (<0.7)	0 (0)	<5 (<0.6)	<5 (<0.7)	<5 (<0.3)	10 (0.3)	5 (0.1)	6 (0.1)	<5 (<1)	0 (0)	0 (0)	0 (0)	<5 (<2.7)	<5 (<2.2)
Chronic respiratory disease	<5 (<1.3)	0 (0)	5 (0.6)	7 (0.8)	0 (0)	20 (1.0)	7 (0.2)	<5 (<0.1)	18 (0.3)	<5 (<1)	0 (0)	<5 (<1.3)	<5 (<4.1)	0 (0)	<5 (<2.2)
Congenital anemia	<5 (<1.3)	0 (0)	<5 (<0.6)	0 (0)	<5 (<0.7)	8 (0.4)	<5 (<0.1)	<5 (<0.1)	<5 (<0.1)	<5 (<1)	0 (0)	0 (0)	0 (0)	0 (0)	<5 (<2.2)
Necrotizing enterocolitis	0 (0)	0 (0)	0 (0)	6 (0.7)	0 (0)	<5 (<0.3)	<5 (<0.1)	0 (0)	6 (0.1)	<5 (<1)	0 (0)	<5 (<1.3)	<5 (<4.1)	0 (0)	0 (0)
Intraventricular hemorrhage	0 (0)	<5 (<0.7)	<5 (<0.6)	7 (0.8)	<5 (<0.7)	8 (0.4)	6 (0.2)	0 (0)	7 (0.1)	<5 (<1)	0 (0)	0 (0)	<5 (<4.1)	<5 (<2.7)	0 (0)
Retinopathy of prematurity	<5 (<1.3)	0 (0)	<5 (<0.6)	6 (0.7)	0 (0)	11 (0.6)	7 (0.2)	0 (0)	8 (0.1)	<5 (<1)	0 (0)	0 (0)	<5 (<4.1)	0 (0)	<5 (<2.2)
Patent ductus arteriosus	0 (0)	<5 (<0.7)	<5 (<0.6)	12 (1.3)	<5 (<0.7)	7 (0.4)	13 (0.3)	6 (0.2)	12 (0.2)	<5 (<1)	0 (0)	0 (0)	<5 (<4.1)	0 (0)	0 (0)
Transient hyperthyroidism	<5 (<1.3)	0 (0)	0 (0)	0 (0)	0 (0)	<5 (<0.3)	0 (0)	0 (0)	<5 (<0.1)	0 (0)	0 (0)	0 (0)	0 (0)	0 (0)	0 (0)
Transient hypothyroidism	0 (0)	0 (0)	0 (0)	0 (0)	0 (0)	0 (0)	0 (0)	<5 (<0.1)	0 (0)	0 (0)	0 (0)	0 (0)	0 (0)	0 (0)	0 (0)
Conditions >28 days after birth, n (%)															
Lack of expected normal physiological development	29 (7.7)	20 (2.7)	34 (4.4)	48 (5.3)	19 (2.6)	62 (3.2)	226 (5.7)	100 (2.9)	153 (2.6)	39 (7.4)	6 (3.7)	8 (2.1)	13 (11)	7 (3.8)	6 (2.7)
Cardiomyopathy	<5 (<1.3)	<5 (<0.7)	<5 (<0.6)	<5 (<0.6)	<5 (<0.7)	<5 (<0.3)	<5 (<0.1)	<5 (<0.1)	<5 (<0.1)	<5 (<1)	0 (0)	0 (0)	<5 (<4.1)	0 (0)	<5 (<2.2)
Heart transplantation	0 (0)	0 (0)	0 (0)	0 (0)	0 (0)	0 (0)	0 (0)	0 (0)	0 (0)	0 (0)	0 (0)	0 (0)	0 (0)	0 (0)	0 (0)
Atrioventricular block during the entire follow-up, n (%)															
Degree unspecified	<5 (<1.3)	0 (0)	0 (0)	7 (0.8)	0 (0)	<5 (<0.3)	0 (0)	0 (0)	0 (0)	0 (0)	0 (0)	0 (0)	0 (0)	0 (0)	0 (0)
First degree	<5 (<1.3)	<5 (<0.7)	<5 (<0.6)	<5 (<0.6)	0 (0)	<5 (<0.3)	0 (0)	<5 (<0.1)	0 (0)	0 (0)	0 (0)	0 (0)	0 (0)	0 (0)	0 (0)
Second/third degree (total)	<5 (<1.3)	7 (0.9)	12 (1.5)	<5 (<0.6)	<5 (<0.7)	8 (0.4)	0 (0)	0 (0)	<5 (<0.1)	0 (0)	0 (0)	0 (0)	0 (0)	0 (0)	0 (0)
Second/third degree (with pacemaker)	<5 (<1.3)	6 (0.8)	8 (1.0)	0 (0)	<5 (<0.7)	8 (0.4)	0 (0)	0 (0)	<5 (<0.1)	0 (0)	0 (0)	0 (0)	0 (0)	0 (0)	0 (0)
Mortality, n (%)															
Neonatal death (≤28 days)	<5 (<1.3)	<5 (<0.7)	<5 (<0.6)	18 (2.0)	<5 (<0.7)	7 (0.4)	12 (0.3)	6 (0.2)	9 (0.2)	<5 (<1)	0 (0)	<5 (<1.3)	<5 (<4.1)	<5 (<2.7)	<5 (<2.2)
Cumulative deaths until end of follow-up	<5 (<1.3)	≤5 (≤0.7)	5 (0.6)	21 (2.3)	≤5 (≤0.7)	10 (0.5)	21 (0.5)	11 (0.3)	25 (0.4)	<5 (<1)	0 (0)	<5 (<1.3)	<5 (<4.1)	≤5 (≤2.7)	<5 (<2.2)
Follow-up time in years															
Median (IQR)	9.1 (5.2-14.2)	9.8 (5.0-14.9)	8.2 (3.6-13.2)	10.0 (5.6-14.6)	10.6 (6.0-16.2)	8.7 (4.3-14.2)	9.9 (5.5-14.8)	8.9 (4.5-13.6)	8.5 (4.1-13.5)	10.4 (5.3-15.3)	7.6 (3.9-12.5)	6.9 (3.4-11.9)	8.2 (4.3-13.9)	7.6 (4.1-13.1)	9.1 (4.3-14.4)

Note: *SjD*, Sjögren’s disease; *SLE*, systemic lupus erythematosus; *RA*, rheumatoid arthritis; *IIM*, idiopathic inflammatory myopathy; *MCTD*, mixed connective tissue disease; *SD*, standard deviation; *IQR*: interquartile range; *SGA*, small for gestational age; *IUGR*, intrauterine growth retardation. To preserve patient anonymity, values less than 5 were presented as '<5′, and others that may reveal the small number were presented as a range.

*Kwok et al. Outcomes of pregnancies complicated by maternal autoimmune diseases in Denmark, Finland, and Sweden: a multi-national population‐based register study. Am J Obstet Gynecol 2026.*

Preterm birth was reported in 6% to 18% of newborns, while low birth weight occurred in 3% to 17%. The proportion of SGA infants ranged from 5% to 24% across disease groups. An Apgar score below 7 at 1 minute was seen in 4% to 10% of newborns, and in <3% at 5 minutes. Between 9% and 24% of newborns were admitted to the neonatal unit. Within four weeks of birth, congenital anomalies were reported in 3% to 8% of newborns, jaundice in 3% to 11%, respiratory distress in 3% to 10%, birth asphyxia in up to 7%, and hypoglycemia in up to 6%. Neonatal cutaneous lupus was identified in <5 newborns in SjD (in Sweden), SLE (in Denmark), and RA (in all three countries), with none reported elsewhere. During a median follow-up ranging from seven to 11 years, delayed physiological development was observed in 2% to 11% of children across the groups. Child mortality ranged from 0.3% to 2.3%, with most countries reporting <5 deaths per disease group.

### Autoimmune atrioventricular block

As shown in Table 5, second/third-degree autoimmune atrioventricular block was most common in children born to mothers with SjD (0.9% in Finland, 1.5% in Sweden, <1.3% in Denmark), and also observed in those born to mothers with SLE (0.4% in Sweden, <0.7% in Denmark, <0.6% in Finland). Fewer than five cases occurred in children of RA mothers in Sweden, with none in Denmark or Finland, while no cases were reported in IIM or MCTD. Most affected children required pacemakers. Within each country and across all autoimmune disease groups, <5 children had cardiomyopathy and none had heart transplantation.

## Comment

### Principal findings

To our knowledge, this is the first study to provide a comprehensive overview of outcomes in pregnancies complicated by autoimmune diseases (SjD, SLE, RA, IIM, or MCTD) across multiple Nordic countries (Denmark, Finland, and Sweden). The results showed a notable risk of adverse pregnancy outcomes, including preeclampsia, gestational hypertension, FGR, and emergency caesarean section. For neonates, preterm birth, low birth weight, SGA, low Apgar scores, congenital anomalies, and high rates of neonatal unit admission were frequently observed. There was a risk of adverse neurodevelopmental outcome during childhood period. Autoimmune atrioventricular block was reported predominantly in children born to mothers with SjD and SLE.

### Results in the context of what is known

We referred to previously published studies to contextualize our findings regarding the risk of adverse outcomes in pregnant women with autoimmune diseases and their children. For example, the prevalence of preterm birth among the general population in Denmark, Finland, and Sweden between 1990 and 2021 varied between 4.9% and 5.3%;^32^ in the present study, we found a prevalence of at least 6% across all autoimmune disease groups, with the highest prevalence reaching 18% among infants born to women with SLE. Other adverse outcomes examined in this study have also been reported in previous publications investigating the general population of the same three countries, including low birth weight (3%–14% vs. 3%–4% in general population^33^^,^^34^), SGA (5%–24% vs. 2%–14%^34^, ^35^, ^36^), preeclampsia (2%–11% vs. 2%–3%^33^^,^^37^^,^^38^), gestational hypertension (2%–7% vs. 1%–2%^33^^,^^38^), total caesarean section (22%–36% vs. 15%–19%^33^^,^^39^^,^^40^), emergency caesarean section (10%–21% vs. 7%–11%^39^^,^^40^), congenital anomalies (3%–8% vs. 2%–5%^41^, ^42^, ^43^), gestational diabetes (≤5% vs. 1%–5%^33^^,^^37^ in Denmark and Sweden, and 11%–17% vs. 9%–19%^44^ in Finland), and low 5-minute Apgar score (1%–2% vs. 1.3%^33^).

While the current study lacks general population data during the study period for comparison, previous comparative research has reported higher risks of adverse outcomes in pregnancies burdened with autoimmune diseases compared to the general population. A recent study in the US reported that pregnancies complicated by SLE had significantly higher risk of preterm birth, low birth weight, SGA, and caesarean section.^45^ Indeed, the negative impact of SLE on classic pregnancy outcomes has been widely reported,^7^, ^8^, ^9^^,^^46^ but our findings also provide a broader account of maternal complications (e.g., gestational hypertension and diabetes, and placenta-related conditions) alongside neonatal (e.g., congenital anomalies, cutaneous lupus, mortality) and childhood outcomes (e.g., delayed physiological development) from the Nordic perspective. In the current study, preterm birth appeared more common among pregnancies complicated by SLE and MCTD, However, since no formal comparisons were performed across disease groups, this observation should be interpreted with caution. Data from national registers did not permit distinction between spontaneous and medically indicated preterm birth. Although preeclampsia was more frequently reported in these two disease groups, other complications including gestational hypertension and FGR did not show a similar pattern, suggesting there may be additional factors contributing to the higher rate of preterm birth observed. Another common autoimmune disease that has been assessed in pregnant women is RA.^10^^,^^11^^,^^27^ In the Nordic countries, the risk of preeclampsia, preterm birth, and SGA in pregnancies complicated by RA was investigated in Sweden and Denmark in 1994 to 2006,^47^ and in 2006 to 2018 in relation to disease activity.^48^^,^^49^ The present study further incorporates data from Finland and adds more recent insights and additional outcomes, thereby painting a more complete clinical picture for these pregnancies within the Nordic countries. For pregnancies complicated by SjD, a recent meta-analysis reported an elevated risk of multiple adverse outcomes.^6^ However, another recent prospective study conducted in France focusing on a smaller, more homogeneous cohort of primary SjD cases (n=106) found no significant difference in the risk of adverse outcomes compared with matched pregnancies from the general population.^22^ There is a growing body of evidence on adverse pregnancy outcomes associated with IIM and MCTD.^12^^,^^13^^,^^26^^,^^50^^,^^51^ However, extensive comparative analyses assessing the risk of adverse outcomes in these pregnancies are still lacking. Our findings may help enhance clinical awareness within the Nordic setting and offer insights applicable to similar populations and healthcare settings. It should also be noted that, while we observed a notable risk of congenital anomalies across all disease groups, one previous phone interview study conducted in the US found that maternal autoimmune diseases and related treatment are not associated with the majority of birth defects.^52^ Further studies are warranted to better understand the risk of congenital anomalies in these pregnancies.

Women with autoimmune diseases, particularly SjD and SLE, frequently carry anti-Ro/SSA and/or anti-La/SSB antibodies that can cause autoimmune atrioventricular block in their offspring.^15^^,^^53^ The risk of developing autoimmune atrioventricular block requiring pacemaker implantation in children born to mothers with SjD was largely comparable to that previously reported for complete atrioventricular block among pregnancies in anti-Ro-positive women (1%–2%).^54^^,^^55^ The fact that anti-Ro/SSA and/or anti-La/SSB antibodies are present in only a proportion of patients with SjD (60%–100%) and SLE (around 40%)^53^ may partly explain the modestly lower risk observed in this study examining all SjD and SLE pregnancies. In addition, we observed relatively high stillbirth rate in pregnancies with SjD in Finland (1%) and SLE in Denmark (2%). These rates appear much higher than the estimates in general populations of Finland and Denmark (0.21% and 0.29% in 2021, respectively),^56^ and are similar to the stillbirth rates previously reported in pregnancies complicated by SjD^57^ and SLE.^58^ Together, these findings highlight the need for close monitoring of such pregnancies.

### Clinical implications

The substantial risk of maternal, fetal, and childhood adverse outcomes described in the present study underscores an unmet need for tailored care in pregnancies complicated by autoimmune diseases. To mitigate these risks and improve outcomes, specialized prenatal care and a multidisciplinary approach involving obstetricians, rheumatologists, and pediatricians are essential. Preconception counseling and individualized care plans before pregnancy, such as optimization of disease control, medication review, and early antibody screening in alignment with ACR and EULAR guidelines, are critical to reducing risks of events such as preeclampsia and preterm birth.^59^ Once pregnant, enhanced prenatal surveillance should be a priority, with regular monitoring of maternal and fetal health to promptly address complications like congenital anomalies, gestational diabetes and FGR, as well as fetal echocardiography among anti-Ro/La-positive mothers to detect and manage fetal cardiac risks. Additionally, the elevated risk of emergency caesarean deliveries in this population emphasizes the importance of planning for safe delivery, including the development of individualized delivery strategies.

### Strengths and limitations

The strengths of this study include that it employs a large, population-based cohort spanning more than 20 years across three Nordic countries, making the results generalizable within the Nordic countries and potentially beyond. Moreover, the use of high-quality data from Nordic national health registers ensures accurate mother-child linkage and reliable data collection with minimal information bias. Furthermore, this study covers multiple autoimmune diseases (SjD, SLE, RA, IIM, and MCTD) and a wide range of pregnancy outcomes, which together provide a comprehensive description of the clinical landscape of affected pregnancies.

Limitations of this study include that it lacks a formal control group, which prevents direct comparisons between women with and without autoimmune diseases. Nevertheless, we discussed our findings by referencing to prevalence reported in the literature and statistics regarding the general population from the same countries. Secondly, although the Nordic countries share similar healthcare systems, variations in demographics, diagnostic criteria, and clinical practice might still be present. For instance, the prevalence of gestational diabetes in Finland was notably higher than in the other two Nordic countries, a finding consistent with previous studies on the general population. The underlying reason remains unclear, but it highlights the importance of considering such discrepancies when interpreting and applying these findings to other populations or clinical settings. Thirdly, pregnant women diagnosed with multiple autoimmune diseases were not analyzed separately due to sample size limitations. Instead, each woman was categorized based on her earliest diagnosis and therefore may not reflect the true severity of her underlying conditions during pregnancy. Fourthly, while we have analyzed a broad range of outcomes, we were unable to examine miscarriage or early fetal loss because national registers do not capture pregnancies ending before 22 gestational weeks. This may have resulted in underestimation of early adverse pregnancy outcomes, particularly among patients with coexisting antiphospholipid antibodies or secondary antiphospholipid syndrome. Fifthly, temporal changes in diagnostic criteria and treatment practices may have influenced the frequency of adverse outcomes; however, subgroup analyses by calendar period were limited by small cell counts. Sixthly, the study cohort was identified based on ICD-10 codes in national registers, and we do not exclude the possibility of patient misclassification. Besides, we lack data on disease activity, autoantibody subtypes, treatment exposure, and disease duration, which limits our ability to fully describe clinical heterogeneity within the cohort. Lastly, as an observational descriptive study, it cannot establish causal relationships between autoimmune diseases and adverse pregnancy outcomes.

## Conclusions

In conclusion, this study provides a comprehensive overview of health outcomes in pregnancies complicated by autoimmune diseases across multiple Nordic countries. These findings highlight notable risks for both mothers and children, underscoring an ongoing unmet medical need. These insights lay the foundation for improving healthcare support and developing targeted interventions to address the unique challenges faced by pregnant women with autoimmune disorders.

## CRediT authorship contribution statement

**Kelvin HM Kwok:** Writing – review & editing, Writing – original draft, Project administration, Methodology, Formal analysis, Data curation, Conceptualization. **Mika Gissler:** Writing – review & editing, Supervision, Methodology, Formal analysis, Data curation. **Julie H Vendelbo:** Writing – review & editing, Methodology, Formal analysis, Data curation. **Rebecca Zaha:** Writing – review & editing, Methodology, Conceptualization. **Sven Wegner:** Writing – review & editing, Methodology, Conceptualization. **Yangjun Liu:** Writing – review & editing, Writing – original draft, Methodology, Formal analysis, Data curation. **Rafiq Muhammad:** Writing – review & editing, Methodology, Formal analysis, Data curation. **Vedran Stefanovic:** Writing – review & editing, Supervision, Methodology. **Lars H Pedersen:** Writing – review & editing, Supervision, Methodology, Formal analysis, Data curation.

## Declaration of competing interest

KHMK, YL, and RM are employees of Ciencia Research AB when the study was conducted. RZ, SW are employees of Johnson & Johnson. JHV, LHP, MG, and VS have no conflict of interest to disclose.

## Appendix Supplementary materials

Image, application 1

## Footnotes

Supplementary material associated with this article can be found, in the online version, at doi:10.1016/j.xagr.2026.100667.
