# Supplementary material for: Outcomes of pregnancies complicated by maternal autoimmune diseases in Denmark, Finland, and Sweden: a multi-national population‐based register study
Source: AJOG Glob Rep. 2026 Jun 20;6(3):100667. doi: 10.1016/j.xagr.2026.100667 (PMC13383213; doi:10.1016/j.xagr.2026.100667)
Supplement: Supplementary file 1 [file mmc1.docx]

**Supplementary Materials**

**Article title**

Outcomes of pregnancies complicated by maternal autoimmune diseases in Denmark, Finland, and Sweden: a multi-national population‐based register study

**Supplementary Content**

**Supplementary Method.** Description of registers in each country

**Supplementary Table 1**. International Classification of Diseases version 10 (ICD-10) codes for autoimmune diseases

**Supplementary Table 2**. ICD-10 codes for maternal and offspring outcomes

**Supplementary Method.** Description of registers in each country

***Sweden***

1. Patient Register (Jan 1^st^ 2000 – Dec 31^st^ 2021)
   - It collects information on inpatient (since 1987) and outpatient (since 2001) specialist healthcare utilization, including patient admission and discharge dates, and the diagnosis codes (International Classification of Diseases 10^th^ version, ICD-10) and procedure codes associated with each care visit. Medications dispensed within the hospital are coded using Anatomical Therapeutic Chemical (ATC) codes.
2. Medical Birth Register (Jan 1^st^ 2000 – Dec 31^st^ 2021)
   - It contains data on births in Sweden since 1973 with a nationwide coverage, including mothers’ demographic characteristics, disease history, parity, pregnancy and delivery information, and neonatal details. All diagnoses are coded using ICD-10 codes.
3. Cause of Death Register (Jan 1^st^ 2000 – Dec 31^st^ 2021)
   - It records death information for all individuals in Sweden, including the date of death.
4. Prescribed Drug Register (July 1^st^ 2005 – Dec 31^st^ 2021)
   - It has a complete data coverage of dispensed prescribed drugs at pharmacies in Sweden since July 2005, including detailed information on the type of dispensed drug (ATC code), date of dispensation, dosage, and quantity.
5. Swedish Neonatal Quality Register, SNQ (Jan 1^st^ 2001 – Dec 31^st^ 2021, retrospective data collection before 2008)
   - It is a quality register that contains data on infants admitted to neonatal units within 27 days of birth, including disease diagnosis (ICD-10) and neonatal treatment. Data collection started in 2001 and reached a national coverage in 2012. It excludes data from maternity units, pediatric units, or pediatric intensive care units.
6. Swedish Rheumatology Quality Register, SRQ (Jan 1^st^ 2000 – Dec 31^st^ 2021)
   - This quality register was established in 1995 and collects data on healthcare contacts within rheumatology, including the diagnosis, treatment, and clinical and serological test results. We have approved access to data on 2 types of medication treatment from SRQ, namely corticosteroids and hydroxychloroquine (HCQ).

***Denmark***

1. Patient Register (Jan 1^st^ 1995 – Dec 31^st^ 2017)
   - Established in 1977, it contains information on all hospital admissions and discharges, together with the associated diagnoses (ICD-10 code) and medication received (ATC code).
2. Medical Birth Register (Jan 1^st^ 1995 – Dec 31^st^ 2017)
   - It was established in 1973 to collect data related to maternity and neonatal care, including pregnancy outcomes, perinatal treatment, and maternal diagnoses (ICD-10).
3. Cause of Death Register (Jan 1^st^ 1995 – Dec 31^st^ 2017)
   - It collects data on the causes of death together with age, gender and other demographic factors for all persons living in Denmark. The register began operation since 1875, and records were computerized since 1970.
4. Prescription Register (Jan 1^st^ 1995 – Dec 31^st^ 2017)
   - It contains reimbursement-driven records of dispensations of prescribed medicines since 1994, including information on the prescribed drug (ATC code) and number of dispensations.

***Finland***

1. Care Register for Health Care, HILMO (Jan 1^st^ 2000 – Dec 31^st^ 2022)
   - Established in 1967, it contains information on all hospital admissions and discharges, together with the associated diagnoses (ICD-10 code) and medication received (ATC code).
2. Register on Reimbursed Medication (Jan 1^st^ 2000 – Dec 31^st^ 2022)
   - It handles health insurance to provide coverage for sickness-related expenses since 1990s and contains data on reimbursement decisions and the associated ICD-10 diagnoses, prescription of reimbursed medications (ATC), and information on medical expenses.
3. Cause of Death Register (Jan 1^st^ 2000 – Dec 31^st^ 2022)
   - It collects data on the causes of death together with age, gender and other demographic factors for all persons living in Finland since 1936.
4. Medical Birth Register (Jan 1^st^ 2000 – Dec 31^st^ 2022)
   - It was established in 1987 to collect data related to maternity care, obstetrical services, and neonatal care, including pregnancy outcomes, perinatal treatment, as well maternal and newborn diagnoses (ICD-10).

**Supplementary Table 1**. International Classification of Diseases version 10 (ICD-10) codes for autoimmune diseases

| Description | Denmark | Finland | Sweden |
| --- | --- | --- | --- |
| SjD | DM350 | M35.0 | M35.0 |
| SLE | DM32 | M32 | M32 |
| RA | DM05-06 | M05-06 | M05-06 |
| IIM | DM33, DM608-9 | M33, M60.8-9 | M33, M60.8-9 |
| MCTD | DM351A | M35.1 | M35.1 |
| Note: SjD, Sjögren’s disease; SLE, systemic lupus erythematosus; RA, rheumatoid arthritis; IIM, idiopathic inflammatory myopathy; MCTD, mixed connective tissue disease. | | | |

**Supplementary Table 2**. ICD-10 codes for pregnancy and child outcomes

| Description | Denmark | Finland | Sweden |
| --- | --- | --- | --- |
| Pregnancy outcomes | | | |
| Gestational hypertension | O13 | O13 | O13 |
| Gestational diabetes | DO24 | O24 | O24 |
| Proteinuria during pregnancy | DO121 | O12.1 | O12.1 |
| Preeclampsia | DO14 | O14 | O14 |
| Eclampsia | DO15 | O15 | O15 |
| Premature placental abruption | DO45 | O45 | O45 |
| Placenta previa | DO44 | O44 | O44 |
| Preterm premature rupture of membranes | DO42 | O42 | O42 |
| Obstetric embolism | DO88 | O88 | O88 |
| Intrauterine growth retardation | DO365 | O36.5 | O36.5 |
| Child outcomes | | | |
| Congenital malformations | DQ00-99 | Q00-99 | Q00-99 |
| Cutaneous neonatal lupus | DL93 | L93 | L93 |
| Neonatal jaundice | DP59 | P59 | P59 |
| Respiratory distress syndrome in newborns | DP22 | P22 | P22 |
| Infection | DA00-DB99 | A00-B99 | A00-B99 |
| Birth asphyxia | DP21 | P21 | P21 |
| Meconium aspiration in newborns | DP240 | P24.0 | P24.0 |
| Chronic respiratory disease during perinatal period | DP27 | P27 | P27 |
| Transient neonatal hypoglycemia | DP704 | P70.4 | P70.4 |
| Neonatal anemia | DP613-4 | P61.3-4 | P61.3-4 |
| Necrotizing enterocolitis in fetus and newborn | DP77 | P77 | P77 |
| Intraventricular hemorrhage | DP520-3 | P52.0-3 | P52.0-3 |
| Retinopathy of prematurity | DH351 | H35.1 | H35.1 |
| Patent ductus arteriosus | DQ250 | Q25.0 | Q25.0 |
| Transient hyperthyroidism in newborns | DP721 | P72.1 | P72.1 |
| Transient hypothyroidism in newborns | DP722 | P72.2 | P72.2 |
| Lack of expected normal physiological development | DR62 | R62 | R62 |
| Cardiomyopathy | DI42 | I42 | I42 |
| Heart transplantation (procedural code) | KFQA | FQA | FQA |
| Atrioventricular block (degree unspecified) * | DI443 | I44.3 | I44.3 |
| Atrioventricular block (first degree) * | DI440 | I44.0 | I44.0 |
| Atrioventricular block (second/third degree) * | DI441-2 | I44.1-2 | I44.1-2 |
| Pacemaker implantation | BFC, DT821, DZ95, KFPF | Z45, Z95 | Z45, Z95 |
| * An individual with autoimmune atrioventricular block is defined as having a diagnosis of atrioventricular block (with the specified ICD codes) and without any congential malformations of the circulatory organs (Q20-28, except for Q24.6). Individuals diagnosed with multiple degrees of atrioventricular block were classified based on the highest degree of block they have, ensuring they are categorized only once under the most severe form. | | | |
